# Clinical Performance of the Spot Vision Photo Screener before and after Induction of Cycloplegia in Children

**DOI:** 10.1155/2019/5329121

**Published:** 2019-04-11

**Authors:** Konuralp Yakar

**Affiliations:** Ataturk State Hospital, Department of Ophthalmology, Sinop, Turkey

## Abstract

**Aim:**

To compare the clinical performance of the Spot Vision Screener used to detect amblyopia risk factors (ARFs) in children before and after induction of cycloplegia; the children were referred because they met the screening criteria of the American Association for Pediatric Ophthalmology and Strabismus (AAPOS).

**Methods:**

The Spot Vision Screener and a standard autorefractometer were used to examine 200 eyes of 100 children aged 3–10 years, before and after cycloplegia induction, in terms of ARFs. Sensitivity, specificity, and positive and negative predictive values for the detection of significant refractive errors were measured using the AAPOS referral criteria. It was explored that Spot Screener data were affected by cycloplegia. The extent of agreement between cycloplegic/noncycloplegic photoscreening data and cycloplegic autorefraction measurements was assessed using Wilcoxon and Spearman correlation analyses.

**Results:**

The Spot's sensitivity was improved from 60.9% to 85.3% and specificity from 94.9% to 87.4% with cycloplegia compared to cycloplegic standard autorefractometer results. The positive predictive value of Spot was 75.7%, and the negative predictive value was 90.4% without cycloplegia. With cycloplegia, the positive predictive value of Spot was 63.6% and the negative predictive value was 95.8%.

**Conclusions:**

The Spot Screener afforded moderate sensitivity and high specificity prior to cycloplegia. The sensitivity and negative predictive value improved after induction of cycloplegia. Examiners should be aware of the effects of cycloplegia on their findings.

## 1. Introduction

Cycloplegic refraction reveals the uncorrected refractive status; accommodation is avoided. Cycloplegic status must be considered when correcting refractive errors in children and young adults with high hyperopia and accommodative esotropia [1, 2]. Also, myopia may be overestimated if cycloplegia is not considered [3, 4]. Cycloplegic refraction is the gold standard for assessment of refractive errors [5–8]. Although atropine inhibits accommodation more effectively than do cyclopentolate and tropicamide, the former drug exhibits significant toxicity, potential side-effects, and an extremely long duration of action, restricting practical usage [9]. Many studies have found that cyclopentolate exerts a stronger cycloplegic effect than tropicamide [10–12]; the former agent is thus widely used.

Amblyopia is the leading preventable and reversible cause of monocular vision impairment in children; the estimated prevalence is 2–5% [13–15]. Amblyopia is classified as refractive, strabismic, deprivational, mixed, or idiopathic [16]. Cycloplegic retinoscopy is widely used to measure refractive errors and prevent refractive amblyopia in children. However, retinoscopy is time-consuming, examiner-dependent, and associated with a steep learning curve [17]. In 2012, the American Academy of Pediatrics, the American Association for Pediatric Ophthalmology and Strabismus (AAPOS), and the American Association of Certified Orthoptists (AACO) recommended instrument-based early pediatric vision screening [18]. In 2013, the AAPOS published guidelines for screening of amblyopia risk factors (ARFs) [19]. The iSee (Ivey Special Eye Examination) Vision Screening Research Program of Canada described the photoscreening-based vision test results of 1,443 preschool children aged 18–59 months [20].

Photoscreening/photorefraction uses an infrared camera to obtain reflected (red) reflex images of the pupils. The Spot Vision Screener (Welch Allyn, Skaneateles Falls, NY, USA; firmware ver. 3.0.02.32, software ver. 3.0.04.06) that was used in this study explores refraction status by recording the reflexes of both pupils simultaneously. It is a noninvasive, handheld, touchscreen, portable rechargeable device. The measuring range is ±7.50 diopters (D) for spherical errors and ±3.00 D for cylindrical errors. The device warns the examiner about significant refractive errors, anisometropia, anisocoria, and strabismus.

With the use of Spot Screener in our department for screening pediatric cases, we observed that it underestimates some hyperopic cases without cycloplegia. There were several patients who had normal results without cycloplegia by Spot, but their parents or/and siblings had spectacles of high diopter hyperopia. After induction of cycloplegia, these cases were noticed to have also high hypermetropia.

Here, this study compared the cycloplegic and noncycloplegic clinical performance of the Spot Screener in terms of detecting ARFs in one hundred Turkish children aged 3–10 years, based on the 2013 AAPOS guidelines.

## 2. Subjects and Methods

Written informed consent was obtained from all parents. This prospective study was performed in accordance with the Declaration of Helsinki and was approved by the Ethics Committee of Ondokuz Mayis University, Samsun, Turkey.

We included 200 eyes of 100 patients aged 3–10 years who visited the Ataturk State Hospital ophthalmology clinic for routine eye examinations. Twenty-three children aged 3–5 years, 50 children aged 6–8 years, and 23 children aged 9‐10 years participated in this study. The exclusion criteria were any history of intraocular surgery, premature retinopathy or medium opacity, congenital cataracts, nystagmus, eccentric fixation, and non-cooperation. All children underwent complete ophthalmological and orthoptic evaluations. Refractive measurements were first obtained using a standard autorefractometer (ARK-1; Nidek, Tokyo, Japan) and then employing the Spot Vision screener. Next, cycloplegia was induced by adding drops of 1% cyclopentolate at 5-min intervals (three drops in total, at 0, 5, and 10 min); 45 min later, all measurements (both devices) were repeated. All measurements were performed by the same technician and all examinations by the same ophthalmologist. Cycloplegic and noncycloplegic Spot Screener results of spherical (S), cylindrical (C), and spherical equivalent (SE) values were compared to the cycloplegic refractions obtained using the fixed autorefractometer. Spherical equivalent was calculated as SE = S + C/2. Vector presentation of cylindrical power enounced as **J0** and **J45** calculated by the following formulas, **J**0=(−*C*/2) *∗* cos(2 *∗* *θ*); **J**45=(−*C*/2) *∗* sin(2 *∗* *θ*). Manufacturer's reference values of the Spot Screener were not used in order to compare the current study's findings with previous studies. The referral criteria of the 2013 AAPOS guidelines for ARF evaluation were used (Table 1).

## 3. Statistics

All statistical analyses were performed using SPSS for Windows version 15.0 (SPSS, Inc., Chicago, Ill.). First, the data were checked for normality using the Kolmogorov–Smirnov test. If the significance value of the test was below 0.05, the data were assumed to have a nonnormal distribution. Since the continuous variables in this study were not normally distributed, they were presented as median and range (minimum value, maximum value). Categorical variables are presented as numbers and frequencies. Frequencies were compared using Pearson's chi-square test. Comparisons between the measurements were performed using Wilcoxon signed-rank test and Spearman's correlation analysis. A *p* value of < 0.05 was assumed to indicate statistical significance.

## 4. Results

We examined 200 eyes of 100 children, of whom 49 (49%) were female and 51 (51%) were male; the median age was 7 years (range: 3–10 years). The fixed autorefractometer data were as follows: median cycloplegic spherical value was +1.25 D (range: –3.25 to +7.5 D); median cylindrical value was –0.50 D (range: –3.50 to +3.50 D), median value of **J0** vector was 0.21 (range −1.29 to 1.64), median value of **J45** vector was 0.0 (range −0.56 to 0.74), and median spherical equivalent was +1 D (range: –3.5 to +7.38 D) (Table 2). Based on the 2013 AAPOS referral criteria, ARFs were detected in 20.5% of children (*n*=41). The most common ARF detected via cycloplegic autorefraction was hypermetropia (9.5% (*n*=19)), followed by astigmatism (6% (*n*=12)) and myopia (5% (*n*=10)).

**Figure 1:**
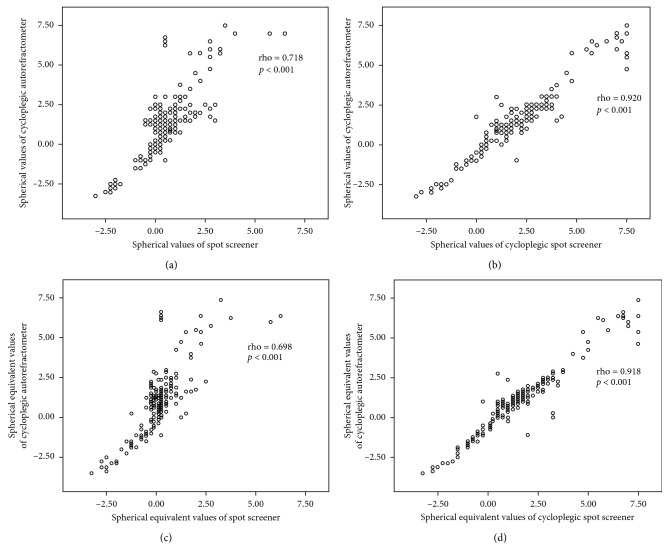
Correlations between spherical and spherical equivalent values of cycloplegic autorefractometer and Spot with or without cycloplegia.

**Figure 2:**
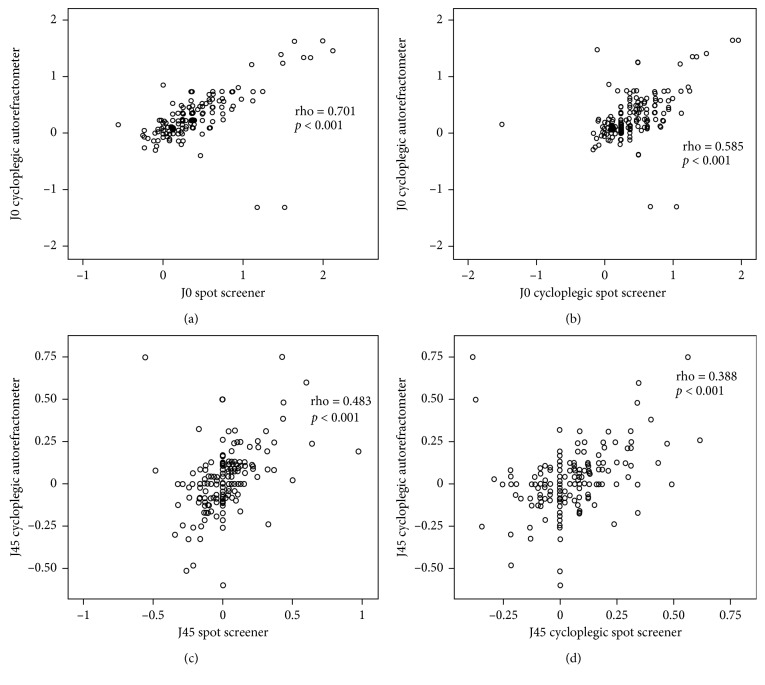
Correlations between **J0** and **J45** values of cycloplegic autorefractometer and Spot with or without cycloplegia.

“In the absence of cycloplegia, the Spot Screener data were as follows: median spherical value was +0.50 D (range:−3 to +6.50 D); cylindrical value was −0.5 D (range: −3 to 0 D), median value of J0 vector was 0.24 (range −0.56 to 2.12), median value of J45 vector was 0.0 (range −0.55 to 0.97), and median spherical equivalent was +0.25 D (range: −3.25 to +6.25 D) (Table 2). ARFs were detected in 27% (*n* = 54) of patients. The cycloplegic data were as follows: median spherical value was +1.75 D (range: −3 to +7.50 D), cylindrical value was −0.75 D (range: −3 to 0 D), median value of J0 vector was 0.21 (range −1.29 to 1.64), median value of J45 vector was 0.0 (range −0.56 to 0.74), and median spherical equivalent was +1 D (range: −3.5 to +7.38 D) (Table 2).” ARFs were detected in 27.5% (*n*=55) of patients. The spherical (rho = 0.718; *p* < 0.001), cylindrical value (rho = 0.706; *p* < 0.001), and spherical equivalent (rho = 0.698; *p* < 0.001) measurements obtained via noncycloplegic Spot screening correlated strongly with the cycloplegic autorefractometer data (*p* < 0.001). The spherical (rho = 0.920; *p* < 0.001), cylindrical value (rho = 0.640; *p* < 0.001), and spherical equivalent (rho = 0.918; *p* < 0.001) measurements of cycloplegic autorefractometer correlated strongly with the cycloplegic Spot Screener data. **J0** vector of cycloplegic autorefractometer was strongly correlated with noncycloplegic Spot (rho = 0.701, *p* < 0.001) and cycloplegic Spot **J0** calculations (rho = 0.585, *p* < 0.001). **J45** vector of cycloplegic autorefractometer was also significantly correlated with noncycloplegic Spot (rho = 0.483, *p* < 0.001) and cycloplegic Spot **J45** calculations (rho = 0.388, *p* < 0.001). Correlations between measurements and power vectors of cycloplegic autorefractometer and Spot Screener are summarized in Figures 1 and 2.

There is a significant difference between the measurements of cycloplegic autorefraction and Spot Screener with and without cycloplegia. All statistics results are summarized in Table 3.

The Spot Screener sensitivity was 60.9% and the specificity was 94.9%, for noncycloplegic measurements. The cycloplegic sensitivity was 85.3%, and the specificity was 87.4%. The noncycloplegic positive predictive value was 75.7%, and the negative predictive value was 90.4%. The cycloplegic positive predictive value was 63.6%, and the negative predictive value was 95.8% in detecting ARFs according to 2013 AAPOS referral criteria.

## 5. Discussion

Most amblyopia is preventable and reversible; this common cause of visual impairment can be reduced by early diagnosis in childhood. Most amblyopia is attributable (completely or partially) to refractive error [15, 21]. The commonly recognized refractive ARFs refer to cycloplegic refractive data, but most vision-screening devices estimate noncycloplegic refractive errors [19]. Noncycloplegic assessments using standard autorefractometers in children and young adults reveal more myopic than cycloplegic refraction, overestimating the incidence/prevalence myopia and underestimating those of emmetropia and hyperopia compared to retinoscopy performed in the cycloplegic state [22].

Peterseim et al. reported that the Spot (Pedia Vision) and Plusoptix A09 (Plusoptix, Inc.) photoscreeners underestimated hyperopia and overestimated myopia in the absence of cycloplegia in children of mean age 6.0 ± 3.4 years [23]. In the present study, the Spot Screener afforded 60.9% sensitivity and 94.9% specificity in the absence of cycloplegia, compared to standard cycloplegic autorefractometer results. The positive predictive value was 75.7% and the negative predictive value 90.4%. On induction of cycloplegia, the sensitivity was 85.3%, the specificity 87.4%, the positive predictive value 63.6%, and the negative predictive value 95.8%.

Peterseim et al. compared also noncycloplegic Spot Screener (ver. 2.0.16) data with cycloplegic retinoscopy findings in 444 children of average age 72 months (range 11–221 months) [24]. The sensitivity was 84.8%, the specificity 70.9%, the positive predictive value 78.1%, and the negative predictive value 79.2%. Arana Mendez et al. compared the same screener with cycloplegic retinoscopy in 219 Costa Rican children aged 20–119 months [25]. The sensitivity was 92.6%, the specificity 90.6%, the positive predictive value 58.1%, and the negative predictive value 98.9%. Forcina et al. tested the same device in 184 children aged less than 3 years (6–35 months) [26]. The screener afforded 89.8% sensitivity, 70.4% specificity, a positive predictive value of 58.9%, and a negative predictive value of 93.6%. All cited studies used the 2013 AAPOS referral criteria for ARFs, as did the current study. In the absence of cycloplegia, the three studies (using the same Spot device) reported different results, reflecting differences in patient age, numbers, and racial profiles. Marzorlf et al. used Spot Screener (ver. 2.1.4) to evaluate 100 children of average age 5.7 years (range 2.2–9.2 years) with developmental disabilities [27]. The sensitivity was 84% and the specificity 62%, thus better than the values of cycloplegic retinoscopy. The positive predictive value was 58% and the negative predictive value 86%. Mu et al. reported a sensitivity value of 94.79% and a specificity value of 85% for Spot Screener (version missing) in detection of amblyopia risk factors in Chinese population within the age group of 4 to 7 years [28]. They also used cycloplegic retinoscopy as gold standard method and AAPOS referral criteria. In a study by Qian et al., compared with cycloplegic retinoscopy, the Spot Screener (v 2.1.4) performed 94.0% sensitivity and 80% specificity without cycloplegia in a cohort of Chinese children aged between 4 and 6 years. Strabismus was also investigated as an ARF in this study, so 65 out of 113 children (57.5%) were found to have at least one ARF. They also pointed out a strong agreement between Spot and retinoscopy [29]. Only refractive errors were investigated in the current study, and refractive ARFs were detected in 20.5% of subjects by cycloplegic autorefraction.

Kirk et al. used Spot Screener (Pedia Vision) and Plusoptix S12 to calibrate and validate the 2WIN photoscreener (Adaptica, Padova, Italy) [30]. They published Spot sensitivity 78%, specificity 59% according to instrument referral criteria in a population of 62 children (age 1 to 10 years; mean 5.2 years).

Refractive status and amblyopia risk factors with autism spectrum disorder (ASD) in 168 Chinese children population aged between 3 and 8 years were compared to age-matched healthy subjects by Wang et al. [31]. Noncycloplegic Spot Screener (version missing) was the only method for detecting ARFs according to AAOPS 2013 referral criteria. Spherical diopter, cylindrical diopter, spherical equivalence, and **J0** and **J45** power vectors were similar between ASD group and controls. Astigmatism (10.1%) was the leading refractive ARF, and strabismus (16.1%) was the most common ARF in ASD group.

Teberik et al. compared the results of three noncycloplegic handheld photorefractometers (Plusoptix A12, Retinomax K-plus 3, Spot Vision Screener version 2.0.16) with those obtained from cycloplegic standard autorefractometer (Topcon KR-8100) in 119 subjects aged between 6 and 17 years [32]. They noticed that Spot Screener performed a statistically significant agreement with Topcon KR-8100 for right eyes' spherical, cylindrical and left eyes' spherical equivalent measurements. The degree of this harmony was declared as moderate. Specificity, sensitivity, and positive/negative predictive values of the Spot in detecting ARFs were not reported.  Table 4 summarizes the results of current and other earlier studies and compares the data.

The newest version of Spot Screener (firmware: 3.0.02.32, software: 3.0.04.069) seems to be less sensitive but more specific than earlier versions under noncycloplegic conditions. After cycloplegia induction, the positive predictive value fell slightly but the negative predictive value rose; the sensitivity (85.3%) and specificity (87.4%) were acceptable. It seems that accommodation is still a standing problem at photoscreeners' existing technology. The Spot analyses the red reflex as well as refractive status better when the pupils are dilated. The differences between our present study and earlier studies may be explained by the methods employed. Cycloplegic autorefraction served as the gold standard in our present study because retinoscopy is examiner-dependent and our study cohort was older than 3 years, thus amenable to standard autorefractometer. Also, the number of participants, age, race, and type of refraction error may have affected the results.

Photoscreening technology was shown to be useful in many previous studies, facilitating early detection of amblyopia and ARFs in children [33–36]. As new versions or devices appear, they must be evaluated. We tested the latest version of the Spot Vision Screener before and after induction of cycloplegia. We found that Spot Screener afforded intermediate sensitivity and high specificity in the absence of cycloplegia (compared to autorefraction), but sensitivity increased after induction of cycloplegia; the positive predictive value decreased but the negative predictive value increased. Examiners should be aware that cycloplegia improves Spot sensitivity and the negative predictive value, and may thus prefer cycloplegic testing for selected cases.

To the best of our knowledge, this is the only study to explore the performance of the latest version of Spot Screener in Turkish children before/after induction of cycloplegia in terms of detecting the ARFs of the 2013 AAPOS referral critter.

## 6. Limitations of the Study

Comparing the results of Spot before/after cycloplegia also with cycloplegic retinoscopy could provide additional benefit to the current study. Since the study population is older than three years old and can cooperate with standard autorefractometers, cycloplegic autorefraction was chosen as the gold standard method for detecting ARFs. Teberik et al. also chose standard autorefractometer as the gold standard method while comparing three different photoscreeners [32]. Crescioni et al. used cycloplegic autorefraction of Retinomax K-Plus2 as a gold standard method instead of cycloplegic retinoscopy while investigating the performance of Spot Screener and Plus Optix [37]. Payerols et al. evaluated the performance of PlusOptix A09 by comparing the results of cycloplegic Retinomax and Nidek ARK-530A refractometer [38].

## Figures and Tables

**Table 1:** Ambliyopia risk factors targeted by automated vision screening (2013 AAOPS guideline).

Age (months)	Refractive risk factors targets (cycloplegic refraction)			
	Astigmatism	Hyperopia	Anisometropia	Myopia
12–30	>2.0 D	>4.5 D	>2.5 D	>−3.5 D
31–48	>2.0 D	>4.0 D	>2.0 D	>−3.0 D
>48	>1.5 D	>3.5 D	>1.5 D	>1.5 D
All ages	Nonrefractive amblyopia risk factor targets: manifest strabismus > 8 PD in primary position media opacity > 1 mm			

D: diopters; PD: prism diopters.

**Table 2:** Median values of refractive parameters and power vectors using cycloplegic autorefraction and Spot Vision Screener with or without cycloplegia.

	Cycloplegic refraction			Spot screener			Cycloplegic spot screener		
	Min	Max	Median	Min	Max	Median	Min	Max	Median
S	−3.25	7.5	1.25	−3.0	6.50	0.50	−3.0	7.50	1.75
C	−3.50	0.0	−0.5	−3.00	0.0	−0.50	−3.00	0.0	−0.75
SE	−3.50	7.38	1.0	−3.25	6.25	0.25	−3.25	7.50	1.25
**J0**	−1.29	1.64	0.21	−0.56	2.12	0.24	−1.49	1.96	0.32
**J45**	−0.56	0.74	0.0	−0.55	0.97	0.0	−0.38	0.61	0.0

S: spherical; C: cylindrical; SE: spherical equivalent.

**Table 3:** Comparison of cycloplegic autorefraction with Spot Screener.

	Spherical	Cylindrical	Spherical equivalent
CA vs Ss	*p* < 0.001^*∗*^	*p* < 0.001^*∗*^	*p* < 0.001^*∗*^
CA vs cycloplegic Ss	*p* < 0.001^*∗*^	*p* < 0.001^*∗*^	*p* < 0.001^*∗*^
Cycloplegic SV vs noncycloplegic Ss	*p* < 0.001^*∗*^	*p* < 0.001^*∗*^	*p* < 0.001^*∗*^

CA: cycloplegic autorefraction; Ss: spot screener. Wilcoxon ^*∗*^*p* < 0.001.

**Table 4:** Comparison of previous studies to current study of Spot Screener in detecting ARFs accordingly to 2013 AAOPS criteria.

Study	Device	Age	Sensitivity	Specificity	PPV	NPV	Compared with
*Peterseim*	Spot (v2.0.16)	11–221	84.8	70.9	78.1	79.2	CR
*Arana*	Spot (v2.0.16)	20–119	92.6	90.6	58.1	98.9	CR
*Forcina*	Spot (v2.0.16)	6–35	89.8	70.4	58.9	93.6	CR
*Marzolf*	Spot (2.1.4)	26.4–110.4	84	62	58	86	CR
*Mu*	Spot (version missing)	48–84	94.79	85	—	—	CR
*Qian*	Spot (v 2.1.4)	48–72	94	80	—	—	CR
*Current study*	Spot (v3.0.04.06)	36–120	60.9	94.9	75.7	90.4	CA
	Spot cycloplegic	36–120	85.3	87.4	63.6	95.8	CA

Age: months; PPV: positive predictive value; NPV: negative predictive value; CR: cycloplegic retinoscopy; CA: cycloplegic autorefractometer.

## Data Availability

No data were used to support this study.

## References

[1] Hiatt R. L., Jerkins G. (1983). Comparison of atropine and tropicamide in esotropia. *Annals of Ophthalmology*.

[2] Bujara K., Schulz E., Haase W. (1981). Skiaskopie mit und ohne cycloplegie bei kindern. *Albrecht von Graefes Archiv für Klinische und Experimentelle Ophthalmologie*.

[3] Prabhakar S. K., Prathibha K. S., Angadhi P. A., Singhal A. K., Ara R. R., Naaz A. S. (2015). Cycloplegic influence on the accuracy of autorefractometer in myopic and hyperopic children. *Nepalese Journal of Ophthalmology*.

[4] Zhu Q., Li F., Wang J., Shen L., Sheng X. (2016). Fecal calprotectin in healthy children aged 1–4 years. *PLoS One*.

[5] Li T., Zhou X., Zhu J., Tang X., Gu X. (2018). Effect of cycloplegia on the measurement of refractive error in Chinese children. *Clinical and Experimental Optometry*.

[6] Morgan I. G., Iribarren R., Fotouhi A., Grzybowski A. (2015). Cycloplegic refraction is the gold standard for epidemiological studies. *Acta Ophthalmologica*.

[7] Sun Y.-Y., Wei S.-F., Li S.-M. (2018). Cycloplegic refraction by 1% cyclopentolate in young adults: is it the gold standard? The Anyang University Students Eye Study (AUSES). *British Journal of Ophthalmology*.

[8] Fotedar R., Rochtchina E., Morgan I., Wang J. J., Mitchell P., Rose K. A. (2007). Necessity of cycloplegia for assessing refractive error in 12-year-old children: a population-based study. *American Journal of Ophthalmology*.

[9] Fan D. S., Rao S. K., Ng J. S., Yu C. B., Lam D. S. (2004). Comparative study on the safety and efficacy of different cycloplegic agents in children with darkly pigmented irides. *Clinical and Experimental Ophthalmology*.

[10] Hofmeister E. M., Kaupp S. E., Schallhorn S. C. (2005). Comparison of tropicamide and cyclopentolate for cycloplegic refractions in myopic adult refractive surgery patients. *Journal of Cataract & Refractive Surgery*.

[11] Yoo S. G., Cho M. J., Kim U. S., Baek S.-H. (2017). Cycloplegic refraction in hyperopic children: effectiveness of a 0.5% tropicamide and 0.5% phenylephrine addition to 1% cyclopentolate regimen. *Korean Journal of Ophthalmology*.

[12] Gettes B. C., Belmont O. (1961). Tropicamide: comparative cycloplegic effects. *Archives of Ophthalmology*.

[13] Donahue S. P., Ruben J. B. (2011). US preventive services task force vision screening recommendations. *Pediatrics*.

[14] Webber A. L., Wood J. (2005). Amblyopia: prevalence, natural history, functional effects and treatment. *Clinical and Experimental Optometry*.

[15] Friedman D. S., Repka M. X., Katz J. (2009). Prevalence of amblyopia and strabismus in white and African American children aged 6 through 71 months the baltimore pediatric eye disease study. *Ophthalmology*.

[16] Simons K. (2005). Amblyopia characterization, treatment, and prophylaxis. *Survey of Ophthalmology*.

[17] Guha S., Shah S., Shah K., Hurakadli P., Majee D., Gandhi S. (2017). A comparison of cycloplegic autorefraction and retinoscopy in Indian children. *Clinical and Experimental Optometry*.

[18] Miller J. M., Lessin H. R. (2012). Instrument-based pediatric vision screening policy statement. *Pediatrics*.

[19] Donahue S. P., Arthur B., Neely D. E., Arnold R. W., Silbert D., Ruben J. B. (2013). Guidelines for automated preschool vision screening: a 10-year, evidence-based update. *Journal of American Association for Pediatric Ophthalmology and Strabismus*.

[20] Asare A. O., Malvankar-Mehta M. S., Makar I. (2017). Community vision screening in preschoolers: initial experience using the plusoptix S12C automated photoscreening camera. *Canadian Journal of Ophthalmology*.

[21] Multi-Ethnic Pediatric Eye Disease Study Group (2008). Prevalence of amblyopia and strabismus in African American and Hispanic children ages 6 to 72 months the multi-ethnic pediatric eye disease study. *Ophthalmology*.

[22] Sankaridurg P., He X., Naduvilath T. (2017). Comparison of noncycloplegic and cycloplegic autorefraction in categorizing refractive error data in children. *Acta Ophthalmologica*.

[23] Peterseim M. M. W., Papa C. E., Wilson M. E. (2014). Photoscreeners in the pediatric eye office: compared testability and refractions on high-risk children. *American Journal of Ophthalmology*.

[24] Peterseim M. M. W., Papa C. E., Wilson M. E. (2014). The effectiveness of the spot vision screener in detecting amblyopia risk factors. *Journal of American Association for Pediatric Ophthalmology and Strabismus*.

[25] Arana Mendez M., Arguello L., Martinez J. (2015). Evaluation of the spot vision screener in young children in Costa Rica. *Journal of American Association for Pediatric Ophthalmology and Strabismus*.

[26] Forcina B. D., Peterseim M. M., Wilson M. E. (2017). Performance of the spot vision screener in children younger than 3 years of age. *American Journal of Ophthalmology*.

[27] Marzolf A. L., Peterseim M. M., Forcina B. D. (2017). Use of the spot vision screener for patients with developmental disability. *Journal of American Association for Pediatric Ophthalmology and Strabismus*.

[28] Mu Y., Bi H., Ekure E. (2016). Performance of spot photoscreener in detecting amblyopia risk factors in Chinese pre-school and school age children attending an eye clinic. *PLoS One*.

[29] Qian X., Li Y., Ding G. (2019). Compared performance of spot and SW800 photoscreeners on Chinese children. *British Journal of Ophthalmology*.

[30] Kirk S., Armitage M. D., Dunn S., Arnold R. W. (2014). Calibration and validation of the 2WIN photoscreener compared to the PlusoptiX S12 and the SPOT. *Journal of Pediatric Ophthalmology & Strabismus*.

[31] Wang J., Ding G., Li Y. (2018). Refractive status and amblyopia risk factors in Chinese children with autism spectrum disorder. *Journal of Autism and Developmental Disorders*.

[32] Teberik K., Eski M. T., Kaya M., Ankarali H. (2018). A comparison of three different photoscreeners in children. *Journal of Pediatric Ophthalmology & Strabismus*.

[33] Panda L., Barik U., Nayak S. (2018). Performance of photoscreener in detection of refractive error in all age groups and amblyopia risk factors in children in a tribal district of Odisha: the tribal Odisha eye disease study (TOES) # 3. *Translational Vision Science & Technology*.

[34] Qian X., Li Y., Ding G. (2018). Compared performance of spot and SW800 photoscreeners on Chinese children. *British Journal of Ophthalmology*.

[35] Kinori M., Molina I., Hernandez E. O. (2018). The plusoptix photo screener and the retinomax autorefractor as community-based screening devices for preschool children. *Current Eye Research*.

[36] Reddy S., Panda L., Kumar A., Nayak S., Das T. (2018). Tribal Odisha eye disease study #4: accuracy and utility of photorefraction for refractive error correction in tribal Odisha (India) school screening. *Indian Journal of Ophthalmology*.

[37] Crescioni M., Miller J. M., Harvey E. M. (2015). Accuracy of the spot and plusoptix photoscreeners for detection of astigmatism. *Journal of American Association for Pediatric Ophthalmology and Strabismus*.

[38] Payerols A., Eliaou C., Trezeguet V., Villain M., Daien V. (2016). Accuracy of PlusOptix A09 distance refraction in pediatric myopia and hyperopia. *BMC Ophthalmology*.

