# Corrigendum to “Clinical Performance of the Spot Vision Photo Screener before and after Induction of Cycloplegia in Children”

**DOI:** 10.1155/2019/8079158

**Published:** 2019-09-02

**Authors:** Konuralp Yakar

**Affiliations:** Ataturk State Hospital, Department of Ophthalmology, Sinop, Turkey

In the article titled “Clinical Performance of the Spot Vision Photo Screener before and after Induction of Cycloplegia in Children” [1], the spot measurement range in the Introduction was incorrect. The authors cited this range from the article available at https://tvst.arvojournals.org/article.aspx?articleid=2683874; however, in the spot user manual, the measurement range is written as “measuring range is ±7.50 diopters (D) for spherical errors and ±3.00 D for cylindrical errors.” Therefore, “measuring range is ±7.50 diopters (D) for spherical errors and ±3.50 D for cylindrical errors” should be corrected to “measuring range is ±7.50 diopters (D) for spherical errors and ±3.00 D for cylindrical errors.”

Additionally, in the Results, there was an error in the Spot Screener data. The authors inadvertently entered spherical (S) value of one patient in Spot Screener and in Cycloplegic Spot Screener columns; therefore, the minimum cylindrical (C) values in Table 2 were −4.25 and −4.00. When correcting this error, the range of cylindrical value became −3.0 to 0, which is the range that is also referred by the manufacturer, as the machine cannot give −4.25 value in cyclinderic measurements since it only gives results between −3.0 and 0. Therefore, Table 2 and the second paragraph in the Results should be corrected as follows.

“In the absence of cycloplegia, the Spot Screener data were as follows: median spherical value was +0.50 D (range:−3 to +6.50 D); cylindrical value was −0.5 D (range: −3 to 0 D), median value of J0 vector was 0.24 (range −0.56 to 2.12), median value of J45 vector was 0.0 (range −0.55 to 0.97), and median spherical equivalent was +0.25 D (range: −3.25 to +6.25 D) (Table 2). ARFs were detected in 27% (*n* = 54) of patients. The cycloplegic data were as follows: median spherical value was +1.75 D (range: −3 to +7.50 D), cylindrical value was −0.75 D (range: −3 to 0 D), median value of J0 vector was 0.21 (range −1.29 to 1.64), median value of J45 vector was 0.0 (range −0.56 to 0.74), and median spherical equivalent was +1 D (range: −3.5 to +7.38 D) (Table 2).” These corrections have been applied in place.

## Figures and Tables

**Table 2:** Median values of refractive parameters and power vectors using cycloplegic autorefraction and Spot Vision Screener with or without cycloplegia.

	Cycloplegic refraction			Spot screener			Cycloplegic spot screener		
	Min	Max	Median	Min	Max	Median	Min	Max	Median
S	−3.25	7.5	1.25	−3.0	6.50	0.50	−3.0	7.50	1.75
C	−3.50	0.0	−0.5	−**3.00**	0.0	−0.50	−**3.00**	0.0	−0.75
SE	−3.50	7.38	1.0	−3.25	6.25	0.25	−3.25	7.50	1.25
**J0**	−1.29	1.64	0.21	−0.56	2.12	0.24	−1.49	1.96	0.32
**J45**	−0.56	0.74	0.0	−0.55	0.97	0.0	−0.38	0.61	0.0

S: spherical; C: cylindrical; SE: spherical equivalent.
